# The Mediating Role of a *Xu*-Argument Based Iterative Translation Continuation Task in the Dynamic Relationships Between Translation Learning Anxiety and Foreign Language Learning Proficiency and Translation Strategies

**DOI:** 10.3389/fpsyg.2022.916597

**Published:** 2022-05-30

**Authors:** Sumin Zhang, Yuhong Ren

**Affiliations:** ^1^School of Foreign Languages, Zhejiang Gongshang University, Hangzhou, China; ^2^International Office, Hebei Normal University, Shijiazhuang, China

**Keywords:** iterative translation continuation task, way of thinking, translation learning anxiety, foreign language learning proficiency, translation strategies

## Abstract

Growing interest has been shown in the effects of the *xu*-argument based translation continuation task, which have been mainly explored *via* the linguistic dimension. The current study, using a pretest-intervention-posttest design, investigated the dynamic relationships among translation learning anxiety, foreign language learning proficiency, and English–Chinese translation strategies under an iterative translation continuation task (ITCT) that lasted 13 turns. The results yielded from 134 student translators showed a significant increase in their translation strategies comprehension and production, with those with a medium level of translation learning anxiety and foreign language learning proficiency achieving the most. It also showed that the significant partial mediating effect of translation learning anxiety between foreign language learning proficiency and the production of translation strategies in the pre-test became insignificant in the post-test, and the insignificant correlation between the comprehension and production of translation strategies in the pre-test became significant in the post-test. The dynamic relationships among translation learning anxiety, foreign language learning proficiency, and translation strategies confirmed the mediating role of the ITCT in attenuating the impact of higher level of translation learning anxiety and lower level of foreign language learning proficiency on the comprehension and use of translation strategies, though its effects could be different for student translators with different levels of translation anxiety and proficiency as measured by different assessments.

## Introduction

Different languages have different ways of thinking (Wang and He, 2016; Shen, 2018, 2020; House, 2020), due to which negative transfer can appear in foreign language comprehension and use in translation, especially for those with higher level of foreign language learning anxiety (MacIntyre and Gardner, 1994; Zhang and Wang, 2006; Wang, 2018a). Therefore, it is necessary for translation instructions to apply appropriate translation training approaches to decrease the student translators’ anxiety to comprehend and use translation strategies properly to reconstruct their ways of thinking (Feng, 2010; Wang, 2019). Translation continuation, as a *xu*-argument based translation task, was proposed to “attenuate the student translators’ negative feelings in translation” (Wang, 2016, 2018a, p. 37), and has been proved to be able to boost their general translation performance (Xu, 2016; Zhang, 2019a,b) and their comprehension and use of translation strategies (Zhang et al., 2021). However, the effects of a translation continuation task on translation learning anxiety have not been empirically explored, leaving the relationships between translation learning anxiety and translation strategies in a translation continuation task unverified. Given that target language development is a dynamic system under the interaction of affective and cognitive factors (De Bot et al., 2007; Chang and Zhang, 2021), and that an iterative continuation task can enhance a learner’s foreign language learning proficiency and positive foreign language learning attitudes (Wang, 2018b; Zhang and Zhang, 2019; Zhang, 2021), it is necessary for us to explore whether the improved proficiency and attitudes can decrease a student translator’s translation learning anxiety. Therefore, the present empirical study, using a pretest-intervention-posttest design, analyses the dynamic relationships between translation learning anxiety and foreign language learning proficiency and translation strategies under an iterative translation continuation task (ITCT) in English–Chinese translation, intending to extend translation continuation studies from an affective perspective and to provide implications to translation learning and teaching.

## Research Background

### Foreign Language Learning Anxiety and Translation Learning Anxiety

Foreign language learning anxiety, as one of the most important variables in foreign language learning, is significantly related to the tolerance of foreign language learning ambiguity and flexibility (Horwitz et al., 1986; Zhang and Wang, 2006; Dewaele, 2013). Language use is linked to culture and way of thinking, and different languages have different cultures and ways of thinking (House, 2015, 2020; Wang and He, 2016), due to which foreign language learning can be an ambiguous situation where foreign language learners may suffer from foreign language learning anxiety, affecting almost every aspect of their foreign language learning (MacIntyre and Gardner, 1994; Zhang and Wang, 2006; Kao et al., 2017). Translation, as an inseparable part of foreign language learning, is “an act of re-contextualization” and is “both an interlingual and an intercultural process” during which an intercultural understanding is very crucial because the translators need to achieve functional equivalence between the source language and the target language (House, 2020, pp. 10–12; Wang, 2019). Therefore, one of the goals of translation learning is to train the learners to notice the differences in “discourse orientations in two linguacultures,” and to use various overt and covert cultural filtering strategies creatively and consciously to shift their way of thinking to enter the marked translation process (House, 2015, 2020, p. 16; Tomozeiu et al., 2016; Dong et al., 2019).

However, the stress incurred from the translation of various themes and styles can exert great translation learning anxiety, making the student translators too nervous and exhausted to be flexible and creative to use various translation strategies to shift their way of thinking (van Egdom et al., 2020). Higher levels of anxiety can negatively influence a foreign language learner’s information input, processing, and output, resulting in translation inaccuracy (MacIntyre and Gardner, 1994). Additionally, in contrast to those with lower levels of foreign language learning anxiety, those with higher levels of anxiety tend to have a poor working memory as well as a poor long-term memory, which can negatively affect their words and information storing and retaining in reading (MacIntyre and Gardner, 1991; Sellers, 2000). Consequently, the student translators’ translation performance can be hampered due to the important contribution of reading in foreign language learning and translation (e.g., Anderson, 1994; Sellers, 2000). What’s worse, Jiang and Dewaele (2019) found relatively higher levels of foreign language learning anxiety of Chinese foreign language learners in comparison to the international sample. Critically, it was found repeatedly that higher levels of foreign language learning anxiety could be negatively correlated to a learner’s foreign language learning attitudes and enjoyment (Horwitz et al., 1986; Dewaele, 2013; Dewaele et al., 2019), dampening their learning motivation and achievements (Saito et al., 2018; James et al., 2021). Accordingly, a vicious circle is very likely to occur among a translation learner’s awareness and use of translation strategies, foreign language learning anxiety, and foreign langue learning proficiency (De Bot et al., 2007; Chang and Zhang, 2021).

Despite these, translation strategies teaching and learning are not well-matched, as they fail to lessen the ambiguous situation to decrease the student translators’ translation learning anxiety and to raise their learning motivation (Tomozeiu and Kumpulainen, 2016). Quite a few studies have explored how an individual learner’ foreign language learning anxiety changes over time through intervention (cf. Gregersen et al., 2014; Saito et al., 2018), how it interacts with a student’s overall L2 development and enjoyment (cf. Dewaele, 2013; Li et al., 2018). However, there is a paucity of research on whether an individual learner’s translation learning anxiety can be changed through translation training. There is also a paucity of research on the interaction between translation learning anxiety and translation strategies development which is important for the translators to shift their way of thinking to improve their translation performance (Dong et al., 2019; House, 2020; Zhang et al., 2021). Therefore, a teaching approach with higher efficiency is called for in order to decrease the foreign language learning ambiguity, to lessen the translation learning anxiety, and accordingly, to increase the student translators’ awareness and use of translation strategies.

### Xu-Argument and the Xu-Argument Based Translation Continuation Task

*Xu*-argument, as a newly emerging view on language learning initiated by Wang (2016), has received considerable attention largely because “it offers a new approach to enhancing pedagogical and methodological efficiency in L2 teaching and learning” (Wang, 2021. p. 2). The origin of the *xu*-argument can be dated back to the 1990s, and based on the *xu*-argument, various continuation tasks have been designed to couple input and output in a rich interactive context, such as integrated reading-writing continuation tasks and integrated reading-translating continuation tasks, among others (Wang, 2016; Zhang and Zhang, 2021). The continuation tasks are characterized by the capitalizing on the asymmetry between language comprehension and production, and the maximizing of the alignment effects in a rich interactive context. The *xu*-argument contends that, *via xu* (continuation) or CEC (completion, extension, and creation) language is learned and high efficiency in language learning is achieved (Wang, 2021), and has been verified by a substantial body of research (e.g., Wang and Wang, 2015; Michell and Cappellini, 2019; Zhang and Zhang, 2021).

The translation continuation, as one of the important *xu*-based continuation tasks, has been verified a highly efficient translation training approach by substantial research (e.g., Xu, 2016; Zhang, 2019a,b; Zhang et al., 2021). Wang (2018a, p. 37) elaborated eight steps to operate the translation continuation task: (i) Select a translation material. Based on the student translator’s target language level, select a bilingual material, and then choose a part of it, the length of which should be no less than 1,000 words. Whatever theme and genre it maybe, it must be a professional translation with high quality; (ii) divide the chosen bilingual material into two parts. The former part should account for about two-thirds of the length, and the latter about one-third. The former part will be used for comparative reading between the source language and the target language, and the latter will be used for continuation translation with only the source language retained; (iii) comprehend the former part. Without referring to the translation, read and comprehend fully the content and the style of the former part; (iv) translate the former part from memory. After full comprehension of the former part, try to translate it from memory, marking the difficult points; (v) compare the source language of the former part with its translation. Read thoroughly the translation of the former part, and then read sentence by sentence to compare the source language with its translation carefully, paying special attention to the difficult points while tasting and appreciating the translation styles used to deal with the differences between the source language and the target language in their way of expressions (the efficacy can be better with a teacher’s guidance); (vi) continue to translate the latter part. Translate the latter part, trying to imitate the styles of the professional translation in the former part. After the first draft, try to revise it repeatedly to make the self-translation of the former part be in line with the other-translation (professional translation) to achieve linguistic and functional equivalence; (vii) compare the self-translation with the other-translation of the latter part, identifying gaps so as to know how to polish the self-translation; (viii) continue to choose another part from the selected bilingual material to repeat the above steps to do iterative continuation translation if you are interested.

In addition to the provision of a certain kind of linguistic and cultural background scaffold, the translation continuation can also help the student translators to find out how the expert use various translation strategies to shift his or her way of thinking to achieve intercultural understanding and functional equivalence (House, 2015; Zhang et al., 2021). The ability to comprehend and use a target language determines the quality of translation, thus, the infusion of *xu* between the target language’s comprehension and use can function as a scaffold to help the student translators to achieve proper mapping between the static language and the dynamic extension in their own translation (Wang, 2020). The proper mapping can, accordingly, lessen the foreign language learners’ affective filter and cognitive load, thus facilitating their cultural filtering through various linguistic means and enhancing their translation performance (Piller, 2017; House, 2020; Wang, 2020). Creative production relates to a foreign language learner’s agency under the interaction of the linguistic and social environment (Shanker and King, 2002), thus, the increased comprehension and production of translation strategies can, in turn, boost their agency to have more foreign language learning motivation, cognition, and emotion in a virtuous circle (Dörnyei, 2019).

The ITCT is an enhanced form of the translation continuation task featuring ergonomics because the student translators can observe and imitate professional translations with different styles and themes in a continued context iteratively (Wang, 2018a; Şahin and Kansu-Yetkiner, 2020; Zhang et al., 2021). In order to further enhance the interaction, Wang (2018b) designed *xu*-based iterative continuation tasks ranging from two turns of coupled reading and writing to a novel with successive turns. One gap in the *xu*-based iterative continuation research is that the majority of the studies conducted so far, to our knowledge, have focused on writing, paying very little attention to the iterative continuation translation, from which an enhanced translation training pedagogy and methodology could be formed (Wang, 2018b). Another gap is that although the continuation translation task is proposed to be able to reduce the student translators’ translation learning anxiety level, and accordingly to activate their awareness and use of the translation strategies in their translation (Wang, 2018a; Zhang et al., 2021), there is a lack of empirical studies to investigate how the ITCT can affect a student translator’s translation learning anxiety, leaving the effects of the ITCT on affective factors like translation learning anxiety and the relationships between translation learning anxiety and translation strategies still unverified.

### Foreign Language Learning Proficiency

Foreign language learning proficiency is another important factor in foreign language processing which has yielded unanimous results in bilingual research. Some studies found that linguistic information processing, such as agreement morphology (Coughlin and Tremblay, 2012), wh-extractions (Zhou et al., 2017), English tense and aspect (Yao and Chen, 2017), Chinese SV and VO dependence structures (Hao et al., 2021), and motion events processing (Zhang, 2021) are significantly influenced by a learner’s foreign language learning proficiency, and that only those with a higher proficiency can process foreign lexical, syntactic, and semantic information as a native. However, some emotional information studies such as Chen et al. (2015); Ivaz et al. (2016), and Iacozza et al. (2017) regard emotional information as fully automatic and unconscious, and irrelevant to foreign language learning proficiency. Additionally, Castillejo (2019) also found proficiency was not a strong predictor of language fluency. We can see from the above mentioned that foreign language learning proficiency might have a different effect on different information processing for it “might not depend on its grammatical formation, but on other relevant factors such as plausibility, embedding, and processability” (Sílvia, 2020, p. 1).

Up to now, the relationships between foreign language learning proficiency and translation strategies comprehension and production has been rarely explored. Different from other skills learning such as listening, speaking, reading, and writing, translation needs the student translators to comprehend and use translation strategies to shift their way of thinking (Feng, 2010; Wang, 2019; House, 2020). Chinese and English have different ways of thinking, which is prone to incur a negative L1 transfer for the Chinese–English translation (Wang and He, 2016; Shen, 2018, 2020), whereas the conscious use of translation strategies is a mark of a higher stage of translation, which can help the student translators to shift their way of thinking to empower their cultural filtering in translation (Xu and Luo, 2006; House, 2015; Piller, 2017). Therefore, it is necessary to analyze whether a student translator’s foreign language learning proficiency can affect their comprehension and production of translation strategies. Given that foreign language learning proficiency and foreign language learning anxiety have been proved to be negatively correlated (Horwitz et al., 1986; Zhang and Wang, 2006; James et al., 2021), it is also necessary to analyze the relationships between foreign language learning proficiency, translation learning anxiety, and translation strategies.

In sum, previous research on translation learning anxiety, translation strategies comprehension and production, and foreign language learning proficiency has indicated relatively close relationships among the three. It also indicates that the ITCT may be able to mediate the relationships among the three. However, to date, the dynamic relationships between translation learning anxiety and translation strategies comprehension and production and foreign language learning proficiency have not been explored. Therefore, the present study investigated whether there could be some changes in the correlations among the three under a *xu*-argument based ITCT. This strand of research can provide a new evidence for the efficacy of the ITCT and a dynamic understanding of the affective and cognitive factors in translation in light of translation learning anxiety and foreign language learning proficiency.

## Research Design

### Research Questions

Q1: Can the ITCT increase the student translators’ comprehension and production of translation strategies to enter the marked conscious translation process? If it is, are there any differences among the student translators with different levels of translation learning anxiety and foreign language learning proficiency?

Q2: Are there some changes in the correlations between translation learning anxiety and foreign language learning anxiety and translation strategies comprehension and production under the ITCT treatment?

### Participants

The participants are 164 Chinese university students enrolled on an English–Chinese translation course. Twenty of them did not answer the questionnaire created to gather participants’ demographic information such as their CET-4 score (a measurement of their foreign language learning proficiency) and learning experience with a ITCT. CET-4 is a Chinese national test for non-English majors which has been proved to have high reliability (Zhang and Wang, 2006; Fan et al., 2018). Nine students did not report their CET-4 scores, and one student did not do the translation production task. Thus, totally 30 students were excluded, leaving 134 participants in the final pool. Each participant was given a small gift for their participation after the treatment.

### Instruments

#### Translation Learning Anxiety Questionnaire

The translation learning anxiety questionnaire was adapted from the Foreign Language Classroom Anxiety Scale invented by Horwitz et al. (1986) which has been used in many studies (Zhang and Wang, 2006; Dewaele, 2013; Castillejo, 2019; Dewaele et al., 2019). According to the present research purpose, original terms, such as “foreign language learning classroom,” “foreign language teacher,” and “foreign language test” were changed to “translation classroom,” “translation teacher,” and “translation test,” respectively. Following Horwitz et al. (1986), a pilot testing with the translation learning anxiety questionnaire was carried out on 39 students from a parallel class in the same university. It demonstrated that students with higher levels of translation learning anxiety could be identified and that they shared a number of characteristics in common, affording an opportunity to examine the scope and severity of translation learning anxiety. The scale demonstrated internal reliability, achieving a Cronbach’s α at 0.846 with all items producing significant corrected item-total scale correlations. Therefore, it is appropriate for the present study and all items were included in the formal investigation, showing an acceptable Cronbach’s α at 0.812 for the whole questionnaire.

#### The Pre-test and the Post-test

The pre-test and the post-test of the translation strategies both contain two parts: the comprehension and production of the translation strategies, each with a total score of 20 points in line with Zhang et al. (2021). The comprehension part includes 10 English–Chinese and 10 Chinese–English bilingual sentences, asking the participants to judge the translation strategies used in each sentence translation according to the 10 translation strategies given. If the participants make a correct judgment, they will be given a score of 1, and a wrong judgment will score 0. The production part contains 10 Chinese sentences and 10 English sentences, asking the participants to do Chinese–English and English–Chinese translation, respectively. If the participants use translation strategies once in their translation, and their translation is also correct, they will be given a score of 1. If the participants use translation strategies, but with an incorrect translation, or no translation strategies is used, their translation will score 0.

In light of the various orientations for translation quality assessment (TQA) provided by translation studies, following a critical analysis of some TQA models (see Lauscher, 2000 for details) as well as House (2015); Wang (2018a), and Dong et al. (2019), the present scoring was done based on the following reasons: (i): comparing the translation with the source text and the parallel text in the target language are two basic approaches to TQA, through which the alignment of the continuation translation with the professional translation could be observed (Lauscher, 2000; Wang, 2018a), (ii) translation process is guided by case-specific values which could be realized by strategies employed and should be “operationalized as evaluation parameters” (Lauscher, 2000, p. 149), and (iii) the purpose of the present study is to see whether the participants can be in a marked translation process to shift their way of thinking which is characterized by conscious use of the translation strategies (Xu and Luo, 2006; House, 2015; Dong et al., 2019).

The translation strategies used were classified and judged according to Feng (2010) and Zhang et al. (2021) by the 2nd author under the instruction of the 1st author of the present study, whose Pearson *r* is 0.950, 0.991, 0.993, and 0.945, respectively. Totally, 10 kinds of translation strategies were categorized: negative-positive shifting, word class shifting, concrete translation, abstract translation, addition or deletion, word splitting or combination, sentence splitting or combination, *trans*-voice translation, transform translation, and degradation or eulogization translation (see Feng, 2010; Zhang et al., 2021 for details). As shown in Example 1, if the student translators translate “evaporation” into “蒸发作用/evaporation function,” or translate “modesty and loftiness” into “谦虚态度和高贵品质/modest attitudes and lofty quality,” it would be labeled with [abstract-concrete shifting]. Also, as shown in Example 2, if the student translators translate “统治/rule (verb)” into “…ruler (noun),” or translate “珍惜/cherish (verb)” into “value (noun),” it would be labeled with [word class shifting]. As shown in Example 3, if the student translators translated “worse” into “没……好到哪去/not better,” or translated “been one of the family” into “不是外人/not an outsider,” it would be labeled with [negative-positive shifting]. Also, as shown in Example 4, if the student translator translated “半个世纪/half a century” into “50 years,” or translated “一百年/100 years” into “…century,” it would be labeled with [transformation translation]. As shown from Example 1 to Example 4, the pre-test and post-test are similar in their difficulty (*r* = 0.940).

Example (1) (a) We should by no means neglect the evaporation of water.

我们不能忽视水的蒸发作用。(concrete translation) (pre-test).

(b) We were all deeply touched by her modesty and loftiness.

我们都被她的谦虚态度和高贵品质所深深打动。(concrete translation) (post-test).

Example (2) (a) 他统治那个地区长达20年之久。

He had been the ruler of that region for as long as 20 years (word class shifting) (pre-test).

(b) 老师教导我们珍惜每一个机会。

The teacher taught us the value of an opportunity (word class shifting) (post-test).

Example (3) (a) I had expected to see a much worse house.

我本来也没指望看到房子好到哪去。[negative-positive shifting] (pre-test).

(b) Osborne had been one of the family anytime these 23 years.

奥巴23年来对这个家庭来说一直就不是外人。[negative-positive shifting] (post-test).

Example (4) (a) 过去的半个世纪见证了中国戏剧的巨大发展。 (pre-test).

The past 50 years [transform translation] witnessed great development in Chinese drama.

(b) 过去的一百年见证了中国的巨大变化。 (post-test).

The past century [transform translation] has witnessed great changes in China.

SPSS23.0 was used to do the analysis, including the paired sample *T*-test, the correlation analysis, and the mediating effect analysis in order to see whether the student translators had made any progress in their translation strategies comprehension and production, whether there were any correlations between foreign language learning proficiency, and translation learning anxiety and translation strategies, and whether there were any changes in the relationships among the three after the ITCT treatment, respectively.

### Training Materials and Procedures

Translation continuation materials were mainly adapted from *Book Worm*, co-edited by Oxford Publishing House and Foreign Language Teaching and Research Publishing House. The topics relate to daily life, science and technology, scientific research, physics, movie comments, literature, education, and political reports, aiming to make the student translators familiar with different themes and styles to meet the requirement of translation featured by super-subjects (Huang and Wang, 2020).

All the training materials were designed according to Wang (2018a) and Zhang et al. (2021). Given that “the efficacy of the translation continuation task can be better with a teacher’s guidance.” Wang (2018a, p. 37), the student translators were instructed to read the first two-thirds of the training material by the 1st author of the present paper in order to make sure the student translators could spot out and appreciate fully the translation strategies used by the expert translator. The remaining one-third of the material was assigned as homework to be translated by the participants, and was checked according to the expert’ translation in the following lesson, during which the instructor tried to lead the participants to compare their translation with the expert’s to comprehend how the experts used strategies to shift their way of thinking.

The whole experiment lasted 13 weeks. The pre-test, together with the translation learning anxiety questionnaire and the demographic information of the participants was carried out in the regular class period in the 1st week, followed by the ITCT treatment and a general review of the translation strategies from the 2nd to the 12th weeks. In the 13th week, the post-test was carried out, which was about 70 min long according to the time taken by the pre-test.

## Results Analysis

### Student Translators’ Comprehension and Production of Translation Strategies Before and After the Iterative Translation Continuation Task

Table 1 shows the statistics analyses of the student translators’ translation learning anxiety, CET-4 test, and comprehension and production of translation strategies before and after the ITCT treatment. In order to investigate whether there are some differences between the pre-test and the post-test for student translators with different levels of translation learning anxiety and proficiency, they were classified into three groups according to their translation learning anxiety and foreign language learning proficiency *M* and *SD*. As shown in Table 1, the mean of the translation learning anxiety is 3.02, with a maximum of 4.24 and a minimum of 1.45, and the mean of the CET-4 is 490.36, with a maximum of 606, and a minimum of 370. Table 1 also shows the comprehension and production of the translation strategies in the pre-test (*M* = 0.10, *Max* = 3, *Min* = 0; *M* = 5.86, *Max* = 11, *Min* = 1), and in the post-test (*M* = 13.37, *Max* = 20, *Min* = 0; *M* = 14.26, *Max* = 20, *Min* = 7). Therefore, those with scores ≤ 3.02 – 0.53, 3.02 + 0.53 > scores > 3.02 – 0.53, or scores ≥ 3.02 + 0.53 were classified into the lower translation learning anxiety group (*n* = 21), medium translation learning anxiety group (*n* = 94), and higher translation learning anxiety group (*n* = 19), respectively. And those with scores ≤ 490.36 – 49.58, 490.36 + 49.58 > scores > 490.36 – 49.58, or scores ≥ 490.36 + 49.58 were classified into the lower foreign language learning proficiency group (*n* = 21), medium foreign language learning proficiency group (*n* = 94), and higher foreign language learning proficiency group (*n* = 19), respectively.

**TABLE 1:** Statistics analyses of the student translators’ TA, CET-4 test, and TS comprehension and production.

	*N*	Min	Max	*M*	*SD*
Translation learning anxiety	134	1.45	4.24	3.02	0.53
CET-4	134	370	606	490.36	49.60
Pre-test comprehension	134	0	3	0.10	0.45
Pre-test production	134	1	11	5.86	1.91
Post-test comprehension	134	0	20	13.37	5.55
Post-test production	134	7	20	14.26	3.45

*TA, translation learning anxiety; CET-4, College English test-4; TS, translation strategies.*

Then, a paired sample *T*-test was used to analysze whether there were some differences between the pre-test and the post-test for student translators with different levels of translation learning anxiety (see Table 2). Table 2 shows a significant improvement of all the student translators in their translation strategies comprehension and production. However, those with a medium level of translation learning anxiety improved the most (*t*_*comprehension*_ = −21.87, *p* < 0.001; *t*_*production*_ = −22.09, *p* < 0.001), followed by those with a lower level of translation learning anxiety (*t*_*comprehension*_ = −13.86, *p* < 0.001; *t*_*production*_ = −13.98, *p* < 0.001) with the higher translation learning anxiety learners facilitated the least (*t*_*comprehension*_ = −10.60, *p* < 0.001; *t*_*production*_ = −9.32, *p* < 0.001). The findings indicated that although the ITCT could increase the student translators’ comprehension and production of translation strategies, its efficacy could be greatest for those with medium level of translation learning anxiety.

**TABLE 2:** Pre–post TS comprehension and production of student translators with different TA.

	TA	Tests	*M*	*SD*	*t*	*p*
Comprehension	Lower	Pre-test	0.14	0.67	−13.86***	0.000
		Post-test	14.57	4.90		
	Medium	Pre-test	0.12	0.44	−21.87***	0.000
		Post-test	12.93	5.61		
	Higher	Pre-test	0.00	0.00	−10.60***	0.000
		Post-test	14.26	5.87		
Production	Lower	Pre-test	6.19	1.89	−13.98***	0.000
		Post-test	15.29	3.42		
	Medium	Pre-test	5.93	1.93	−22.09***	0.000
		Post-test	14.02	3.41		
	Higher	Pre-test	5.16	1.74	−9.32***	0.000
		Post-test	14.32	3.67		

***The significant level is 0.001; TS, translation strategies; TA, translation learning anxiety.

A paired sample *T*-test was also used to analyze whether there were some differences between the pre-test and the post-test for student translators with different levels of foreign language learning proficiency (see Table 3). Table 3 shows a significant improvement of all the student translators in their translation strategies comprehension and production. However, those with a medium level of foreign language learning proficiency improved the most (*t*_*comprehension*_ = 21.97, *p* < 0.001; *t*_*production*_ = 21.84, *p* < 0.001), followed by those with a higher level of foreign language learning proficiency (*t*_*comprehension*_ = 14.17, *p* < 0.001; *t*_*production*_ = 11.40, *p* < 0.001) with the lower foreign language learning proficiency learners facilitated the least (*t*_*comprehension*_ = 10.19, *p* < 0.001; *t*_*production*_ = 11.19, *p* < 0.001). The findings indicated that although the ITCT could increase student translators’ comprehension and production of translation strategies, its efficacy could be greatest for those with medium level of foreign language learning proficiency.

**TABLE 3:** Pre–post TS comprehension and production of student translators with different FLLP.

	FLLP		*M*	*SD*	*t*	*p*
Comprehension	Lower	Pre-test	0.04	0.02	−10.19***	0.000
		Post-test	12.78	5.96		
	Medium	Pre-test	0.13	0.52	−21.97***	0.000
		Post-test	13.50	5.74		
	Higher	Pre-test	0.05	0.22	−14.17***	0.000
		Post-test	13.48	4.29		
Production	Lower	Pre-test	5.30	1.72	−11.19***	0.000
		Post-test	13.83	3.65		
	Medium	Pre-test	5.79	1.85	−21.84***	0.000
		Post-test	14.28	3.54		
	Higher	Pre-test	6.76	2.12	−11.40***	0.000
		Post-test	14.67	2.87		

***The significant level is 0.001; TS, translation strategies; FLLP, foreign language learning proficiency.

The findings in Tables 1, 2 combined answered the first research question: The ITCT could increase the student translators’ comprehension and production of translation strategies to enter the marked conscious translation process, irrespective of their levels of translation learning anxiety and foreign language learning proficiency, though those with medium level of translation learning anxiety and foreign language learning proficiency achieved the most.

### The Dynamic Relationships Between Foreign Language Learning Proficiency and Translation Learning Anxiety and Translation Strategies Comprehension and Production

A complex correlation among foreign language learning proficiency, translation learning anxiety, and translation strategies comprehension and production before and after the ITCT treatment can be seen from the correlation analyses (see Table 4). First, Table 4 shows a significant positive correlation between foreign language learning proficiency and the pre-test translation strategies production (*r* = 0.20, *p* = 0.022), while no significant correlation was found between foreign language learning proficiency and the post-test translation strategies production (*r* = 0.06, *p* = 0.521). Additionally, a significant correlation was found between the pre-test and the post-test in translation skill production (*r* = 0.19, *p* = 0.027), and between the comprehension and the production of translation strategies in the post-test (*r* = 0.72, *p* < 0.001), whereas no significant correlation was found between the comprehension and the production of translation strategies in the pre-test (*r* = −0.04, *p* = 0.676). Table 4 also shows that translation learning anxiety was significantly negative related to foreign language learning proficiency (*r* = −0.17, *p* = 0.05) which was significantly positive related to the pre-test production of translation strategies (*r* = 0.20, *p* = 0.022).

**TABLE 4:** The relationships among TA, FLLP, and the pre–post-test TS comprehension and production.

	TA	FFLP	Pre-c	Post-c	Pre-p	Post-p
TA	1					
FFLP	−0.17*	1				
Pre-comprehension	−0.05	0.01	1			
Post-comprehension	−0.01	0.02	−0.07	1		
Pre-production	−0.16	0.20*	−0.04	0.06	1	
Post-production	−0.08	0.06	−0.03	0.72**	0.19*	1

*The significant level is 0.05; **the significant level is 0.01; FLLP, foreign language learning proficiency; TA, translation learning anxiety; -c, comprehension; -p, production.

The complex correlations among translation learning anxiety, foreign language learning proficiency, and translation strategies comprehension and production make it necessary to further analyze the relationships among them. Therefore, using Multiple-Linear Regression with pre-test translation strategies production as a dependent variable, the CET-4 score as an independent variable, and translation learning anxiety as the mediating variable, the present study made a path analysis.

**FIGURE 1:**
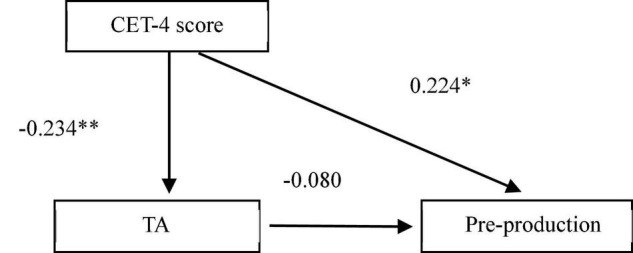
The partial mediating effect of translation learning anxiety (TA). *The significant level is 0.05; **The significant level is 0.01.

Table 5 and Figure 1 show that (i) the student translators’ CET-4 score had a significant influence on their translation learning anxiety (β = −0.234, *t* = −2.762, *p* = 0.007), and (ii) CET-4 had a significant effect on the production of translation strategies in the pre-test both with translation learning anxiety (β = 0.243, *t* = 2.872, *p* = 0.005) and without translation learning anxiety (β = 0.224, *t* = 2.575, *p* = 0.011) with the former more significant. The findings demonstrated that translation learning anxiety played a partial mediating effect between foreign language learning proficiency and the production of translation strategies in the pre-test, indicating an indirect effect of translation learning anxiety on the use of translation strategies in the pre-test. However, in the post-test, no significant correlation was found between the student translators’ foreign language learning proficiency and their use of translation strategies (*r* = 0.06, *p* = 0.52), or between their translation learning anxiety and production of translation strategies (*r* = −0.08, *p* = 0.348). Namely, the significant partial mediating effect of translation learning anxiety between foreign language learning proficiency and the production of translation strategies in the pre-test became insignificant in the post-test.

**TABLE 5:** The partial mediating effect of TA between FLLP and the pre-test TS production.

	Pre-test TS production		
	β	*t*	*p*
The effect of CET-4 on pre-test TS production without TA	0.224*	2.575	0.011
The effect of CET-4 on TA	−0.234**	−2.762	0.007
The effect of TA on pre-test TS production	−0.080	−0.923	0.358
The effect of CET-4 on pre-test TS production with TA	0.243**	2.872	0.005

*The significant level is 0.05; **the significant level is 0.01; FLLP, foreign language learning proficiency; TA, translation learning anxiety; TS, translation strategies.

Although path analysis hasn’t been done due to the insignificant correlation between the student translators’ CET-4 score and their comprehension of translation strategies in both the pre-test (*r* = 0.01, *p* = 0.932) and the post-test (*r* = 0.02, *p* = 0.827), significant changes were found in the correlation between the comprehension and production of translation strategies from the pre-test (*r* = −0.04, *p* = 0.676) to the post-test (*r* = 0.72, *p* < 0.001). Therefore, generally speaking, dynamic relationships were found between translation learning anxiety, and foreign language learning proficiency, and the comprehension and production of translation strategies. The findings answered research question 2, indicating that the ITCT could activate the student translators’ comprehension and conscious use of translation strategies, though its effects could be different to some extent.

## Discussion

### Effects of the Iterative Translation Continuation Task on the Relationships Between Translation Learning Anxiety and Foreign Language Learning Proficiency and the Production of Translation Strategies

The present study provides a new evidence for the efficacy of the *xu*-argument based ITCT through exploring the interaction between affective factors such as translation learning anxiety and cognitive factors such as foreign language learning proficiency. The student translators’ increased comprehension and production of the translation strategies, irrespective of their level of translation learning anxiety and foreign language learning proficiency, confirmed the efficacy of the ITCT as an effective treatment to increase the student translators’ comprehension and conscious use of translation strategies in line with Zhang et al. (2021). The present study also found dynamic relationships between translation learning anxiety and foreign language learning proficiency and the comprehension and production of translation strategies, indicating that the ITCT could inhibit the negative effect of higher translation learning anxiety and lower foreign language learning proficiency in translation, facilitating the student translators to shift their way of thinking in translation. Therefore, the findings are in line with previous studies such as Xu (2016) and Zhang (2019a, 2019b), adding affective evidence to the high efficacy of the ITCT. What needs our special attention is that the present study also found different effects of the ITCT on the comprehension and production of the translation strategies in line with Zhang et al. (2021), which might be due to the relatively less difficult cognitive load in the comprehension than in the production of the translation strategies (Tasseva-Kurktchieva, 2015). Additionally, in line with Dong et al. (2019) who found that interpreting strategies were positively correlated with student interpreters’ interpreting performance, the present findings also verified the efficacy of translation strategies training, confirming that translation, as well as interpreting, could be effectively trained through *xu* which is characterized by a close combination of input and output in a rich interactive context. The present findings also extended the *xu*-argument, verifying that the ITCT could decrease the student translators’ affective and cognitive load (Wang, 2018a), boosting their conscious activation of translation strategies to come to a marked translation stage (Xu and Luo, 2006; House, 2015, 2020).

In addition, in contrast to the former static anxiety and foreign language learning proficiency research (Dewaele, 2013; Dewey et al., 2018; Dewaele et al., 2019; Zhang, 2021), the present study explored anxiety and foreign language learning proficiency dynamically, pointing out that relationships among anxiety, foreign language learning proficiency, and translation strategies could be changed through the ITCT. Previous foreign language learning anxiety research such as Zhang and Wang (2006) and James et al. (2021) found that foreign language learning anxiety was significantly negative related to foreign language learning, and previous foreign language learning proficiency research found significant positive correlations (e.g., Coughlin and Tremblay, 2012; Hao et al., 2021; Zhang, 2021), or insignificant correlations between foreign language learning proficiency and information processing (e.g., Ivaz et al., 2016; Iacozza et al., 2017). Different from the previous studies that investigated the effect of foreign language learning anxiety or foreign language learning proficiency as a static factor, the present study dynamically analyzed the relationships among translation learning anxiety, foreign language learning proficiency, and translation strategies, verifying the mediating role of the ITCT in the effects of the affective and cognitive factors on translation.

### The Reasons for the Facilitative Efficacy of the Iterative Translation Continuation Task

The increased agency of the student translators after the treatment might partly explain the efficacy of the ITCT and its mediating role in the relationships between translation learning anxiety and foreign language learning proficiency and translation strategies. Foreign language learners are dynamic individuals under the interaction of society, psychology, and affection, with a change in one possibly resulting in changes in another (De Bot et al., 2007; Zhang and Zhang, 2021). During the ITCT, the translation learners, under the guidance of the expert’s translation in the previous part, could constantly revise and polish their translation, achieving alignment with the expert’s translation with the enhancement of the interactive factors (Thompson and Valsiner, 2002; Zhang and Zhang, 2021). Therefore, the student translators’ increased translation proficiency might decrease their translation learning anxiety, and accordingly, the raised translation proficiency, together with the lessened translation learning anxiety, might arouse or enhance their greater foreign language learning agency and self-efficacy in an optimal circle (Dörnyei, 2019). Thus, it is natural for the ITCT to bring dynamic changes to the relationships between the student translators’ use of translation strategies and foreign language learning proficiency and translation learning anxiety.

The optimal interactive learning context created by the ITCT also attributes to the student translators’ creative imitation in translation. Creative learning needs two conditions: sustainability and the resources for sustainable development (De Bot et al., 2007). In the ITCT, there is always an asymmetry between the expert’s translation and the student translators’ translation, the sustainability of which can function as one of the engines of the ITCT to push the student translators to align with the expert’s translation, because incomplete input and a gap between the expert’s translation and the student translators’ translation could intimately couple the static learning with the creative use of the translation strategies (Wang, 2018a,^2020^; Zhang, 2019a,b). Additionally, the ITCT can provide the resources for sustainable development, because the close combination of static input and dynamic use in an iterative native-like contextualized interaction tends to make the student translators consciously imitate the expert in their translation to achieve interactional synchrony (Wang, 2020; Chang and Zhang, 2021). Therefore, the present study confirmed the efficacy of the ITCT as an effective approach to nurture the student translators to notice and use appropriately the translation strategies which are vital for the student translators to shift their way of thinking to achieve cultural filtering (Piller, 2017; Wang, 2019; House, 2020).

### The Different Facilitative Efficacy of the Iterative Translation Continuation Task on Student Translators With Different Levels of Translation Learning Anxiety and Foreign Language Learning Proficiency

The unique feature of translation can partly explain the fact that the student translators with medium foreign language learning proficiency and translation learning anxiety could benefit the most from the ITCT. Different from listening, speaking, reading, and writing, translation requires a shift in the way of thinking because of the different cultures and the culture loaded concepts and conceptualization (Wang and He, 2016; Zhang, 2021). Therefore, a qualified translator should be equipped with both linguistic and cross-cultural knowledge, and some abilities to use translation strategies flexibly to shift his or her way of thinking to process the target language appropriately (Tomozeiu et al., 2016; Wang, 2019; House, 2020). On the one hand, the fact that the student translators with medium translation learning anxiety and foreign language learning proficiency were benefited the most might indicate that a proper level of translation learning anxiety can facilitate foreign language learning (Horwitz et al., 1986; Zhang and Wang, 2006). On the other hand, the findings might indicate that foreign language learning proficiency could not be indicative of a student translator’s conscious comprehension and use of translation strategies, because the high correct translation ratio by a higher foreign language learning proficiency might not indicate that the translators had used the translation strategies consciously and purposefully (Feng, 2010; Wang and He, 2016). Some research even found that higher levels of proficiency is not always linked to lower levels of foreign language learning anxiety (Marcos-Llinás and Garau, 2009), and claimed that foreign language reading anxiety was not a specific anxiety for language learning (Sparks et al., 2018). Given the mixed findings in relation to anxiety and the important role of reading in foreign language learning and translation (Sellers, 2000), more research need to be done to further explore the relationship between translation learning anxiety and foreign language learning proficiency and the comprehension and use of translation strategies.

The student translators with medium foreign language learning proficiency and translation learning anxiety benefited the most from the ITCT might also be partly due to the different interactions between the student translators and the ITCT treatment. Low learning efficiency may be due to the inefficient interaction among the main components in a system (Van Geert, 1991, 1994; Wang, 2018b). As for those with a higher foreign language learning proficiency, they may already be equipped with a certain kind of translation strategies, which may make it harder for them to have the same interaction with the ITCT as those with medium foreign language learning proficiency and translation learning anxiety, because the space to be further improved is relatively limited without the sustainability of creative development (De Bot et al., 2007). As for those with rather lower foreign language learning proficiency, they may have a lower foreign language learning ability and be in more need of ITCT treatment, making it relatively difficult for them to have an efficient and sufficient interaction in the ITCT treatment that only lasts a couple of weeks (Wang, 2018b). However, the present study has not measured the student translators’ foreign language learning proficiency after the treatment. Given that a *xu*-based continuation task can also increase the foreign language learners’ foreign language learning proficiency (Wang and Wang, 2015; Zhang and Zhang, 2019), and that all the student translators have made significant progress in their comprehension and production of translation strategies, future studies should be carried out to further analyze whether an increased comprehension and production of translation strategies can result in enhanced foreign language learning proficiency.

## Conclusion

The present study investigated the dynamic relationships among translation learning anxiety, foreign language learning proficiency, and the comprehension and production of translation strategies. It was found that the ITCT could increase the student translators’ TS comprehension and production, though its efficacy could be shown to be greatest for those with a medium level of translation learning anxiety and foreign language learning proficiency. In addition, the present study also found that the ITCT could inhibit the negative effect of higher translation learning anxiety and lower foreign language learning proficiency on the comprehension and production of translation strategies. The findings of the present study provide a new evidence for the *xu*-argument based ITCT, extending the *xu*-argument based research from an affective perspective.

The present findings also have several pedagogical implications and applications. First, in order to maximize the effects of the ITCT, the student translators’ affective factors, such as translation anxiety should be paid attention to by the instructors. Just as Dewaele et al. (2019) and Dörnyei (2019) observed, a better understanding of the learners’ learning experience in light of their positive emotion was closely related to a virtuous learning circle. Therefore, in the application of the ITCT, the instructors are supposed to guide the student translators to lessen their cognitive and linguistic load to increase their self-efficacy, and accordingly to lessen their translation anxiety. Second, as the ITCT has been proved to be able to enhance the student translators’ comprehension and use of translation strategies and have a mediating effects on the relationships between foreign language learning proficiency, foreign language learning anxiety, and translation performance, it is highly suggested that the ITCT be incorporated into general translation instruction to raise the student translators’ “awareness of the factors involved in translation” to empower their self-esteem in translation (Kiraly, 2000, p. 49).

Despite its findings and implications, this study has some limitations. First, it only indirectly investigated the effect of the ITCT on the student translators’ translation learning anxiety through its mediating role between foreign language learning proficiency and translation strategies production. Therefore, further studies should be done to explore the direct effect of the ITCT on translation learning anxiety. Additionally, the present study only investigated the student translators’ comprehension and production of translation strategies, albeit comprehensively, without differentiating Chinese–English translation from English–Chinese translation. Given the differences in translation strategies comprehension and production between Chinese–English translation and English–Chinese translation (Wang, 2019; Zhang et al., 2021), future studies should also differentiate the source and the target language of a certain language in the ITCT. Second, the present study did not consider the changes in the student translators’ foreign language learning proficiency after the ITCT. Target language learning is a dynamic process under the interaction of multiple variables (Chang and Zhang, 2021), and acquisition can take place incidentally when learners comprehend the input. Thus, it is necessary to further investigate whether the present findings were due more to the increased foreign language learning proficiency, or to the lessened translation learning anxiety.

## Data Availability Statement

The original contributions presented in the study are included in the article/supplementary material, further inquiries can be directed to the corresponding author.

## Author Contributions

SZ conceived and designed the study, collected the data, drafted, revised the manuscript, and got it ready for submission. YR collected and analyzed the data under the instruction of SZ. Both authors contributed to the article and approved the submitted version.

## Conflict of Interest

The authors declare that the research was conducted in the absence of any commercial or financial relationships that could be construed as a potential conflict of interest.

## Publisher’s Note

All claims expressed in this article are solely those of the authors and do not necessarily represent those of their affiliated organizations, or those of the publisher, the editors and the reviewers. Any product that may be evaluated in this article, or claim that may be made by its manufacturer, is not guaranteed or endorsed by the publisher.
